# CIRCNV: Detection of CNVs Based on a Circular Profile of Read Depth from Sequencing Data

**DOI:** 10.3390/biology10070584

**Published:** 2021-06-25

**Authors:** Hai-Yong Zhao, Qi Li, Ye Tian, Yue-Hui Chen, Haque A. K. Alvi, Xi-Guo Yuan

**Affiliations:** 1 School of Computer Science and Technology, Liaocheng University, Liaocheng 252000, China; zhaohaiyong@lcu-cs.com; 2 School of Computer Science and Technology, Xidian University, Xi’an 710071, China; q77liqi@163.com (Q.L.); tianye1789470358@163.com (Y.T.); prappo13@gmail.com (H.A.K.A.); 3 Shandong Provincial Key Laboratory of Network Based Intelligent Computing, University of Jinan, Ji’nan 250022, China; yhchen@ujn.edu.cn

**Keywords:** copy number variations, next-generation sequencing, tumor purity, polar coordinate transformation, local outlier factor

## Abstract

**Simple Summary:**

In this study, we propose a copy number variation (CNV) detection method called CIRCNV, which is based on a circular profile of the read depth from sequencing data. The proposed method is an extended version of our previously developed method CNV-LOF. The main difference of CIRCNV from CNV-LOF lies in its two new features: (1) it transfers the read depth profile from a line shape to a circular shape via a polar coordinate transformation to generate a meaningful two-dimensional dataset for CNV analysis and promote fairness between the ends and middle part of the genome, and (2) it performs two rounds of CNV declaration via estimating tumor purity and recovering the truth circular RD profile. We test and evaluate the performance of CIRCNV via conducting simulation studies and real sequencing tumor sample applications. The experimental results show that CIRCNV outperforms peer methods with respect to sensitivity, precision, and the F1-score. The experiments prove that the proposed method is a reliable and effective tool in the field of variation analysis of tumor genomes.

**Abstract:**

Copy number variation (CNV) is a common type of structural variation in the human genome. Accurate detection of CNVs from tumor genomes can provide crucial information for the study of tumor genesis and cancer precision diagnosis. However, the contamination of normal genomes in tumor genomes and the crude profiles of the read depth make such a task difficult. In this paper, we propose an alternative approach, called CIRCNV, for the detection of CNVs from sequencing data. CIRCNV is an extension of our previously developed method CNV-LOF, which uses local outlier factors to predict CNVs. Comparatively, CIRCNV can be performed on individual tumor samples and has the following two new features: (1) it transfers the read depth profile from a line shape to a circular shape via a polar coordinate transformation, in order to improve the efficiency of the read depth (RD) profile for the detection of CNVs; and (2) it performs a second round of CNV declaration based on the truth circular RD profile, which is recovered by estimating tumor purity. We test and validate the performance of CIRCNV based on simulation and real sequencing data and perform comparisons with several peer methods. The results demonstrate that CIRCNV can obtain superior performance in terms of sensitivity and precision. We expect that our proposed method will be a supplement to existing methods and become a routine tool in the field of variation analysis of tumor genomes.

## 1. Introduction

Copy number variation (CNV), as a category of structural variations ranging from several K base pairs (bp) to several M bp or more long, is very common in human tumor genomes. Systematic analysis of CNVs plays an important role in the study of tumor evolution and genesis, as well as cancer treatment and precision diagnosis [1,2]. Accurate detection of CNVs from tumor genomes is the central procedure for this task. The rapid development of next-generation sequencing technologies (NGS) facilitates and promotes the detection of CNVs by providing extremely high-resolution data, and a lot of computational methods have been proposed in recent years. These methods are designed to detect CNVs from individual or multiple samples at the scale of individual chromosomes, whole genomes, or whole exomes [3,4,5,6,7]. By far, a great number of CNV events associated with human cancers have been discovered and are used for deep analysis of cancer mechanisms and treatment. However, in medical practice, accurate detection of CNVs is still challenging due to many factors such as contamination of normal genomes in tumor genomes, absence of normal matched samples, low level of coverage depth, and noisy sequencing data. Currently, there are no existing methods that are versatile enough to detect CNVs accurately when these factors exist at the same time.

From the perspective of sample analysis mode, the existing methods for the detection of CNVs from NGS data could be classified into three categories: multiple-sample-based mode, tumor–normal matched samples-based mode, and single-sample-based mode. The first category of modes is mainly used for the discovery of biologically significant CNV events from human genomes. For example, recurrent CNVs across multiple tumor samples usually confer biological functions to the foundation and progress of cancer cells [8,9,10]. Popular methods of such category include CODEX [11], panelcn.MOPS [12], DCC [7], WaveDec [13], and HetRCNA [14]. The other two categories of modes are primarily used for the detection of CNVs with the purpose of analyzing genetic diversity and seeking out mutated genes in individuals. This could directly contribute to the treatment of cancer patients in medical practice. Tumor–normal matched samples-based methods mainly include GATK (https://gatk.broadinstitute.org, accessed on 10 June 2021), CNV-seq [15], CoNVEX [16], m-HMM [17], CopywriteR [18], CNV-RF [19], EXCAVATOR2 [20], CNVnorm [21], WaveCNV [22], and CNVkit [23]. Such methods have the merit of distinguishing somatic CNVs from germline ones in tumor genomes, but the sequencing cost is relatively high. Single-sample-based methods mainly include CNVnator [24], Control-FREEC [25], VisCap [26], CONDEL [6], CoNVaDING [27], CLImAT-HET [28], iCopyDAV [29], CNV_IFTV [30], CNV-LOF [31], readDepth [32], and GROM-MD [33]. Such methods do not require normal matched samples and can save a piece of the sequencing cost. Moreover, normal matched samples are not usually available in medical practice. Therefore, single-sample-based methods may be preferred in the treatment of patients. Meanwhile, single-sample-based methods can be easily extended to analyze tumor–normal matched samples when the matched samples are obtained. With these considerations, we focus on the mode of single samples in the detection of CNVs from NGS data in this paper.

With the intrinsic characteristics of NGS data, the detection of CNVs based on the depth of coverage usually goes through the following basic steps: (i) data preprocessing (e.g., trimming low-quality reads), (ii) alignment of reads to the latest version of the human genome reference, (iii) calculation of a read count (RC) for each genome position and generation of a read depth (RD) profile for the genome to be analyzed, (iv) correction of GC content bias on the RD profile, and (v) establishing statistical or computational models for analyzing the RD profile in order to call CNVs. The main difference among the existing methods is that they take different viewpoints on the RD profile and adopt different measurements to assess the variance of RD values of genome bins. For example, Control-FREEC [25] looks at the RD profile from a global perspective and takes advantage of the difference between RD values to detect CNVs. Similar methods include readDepth [32], CONDEL [6], and iCopyDAV [29]. One outstanding feature of Control-FREEC is that it is able to utilize the GC content to normalize RD values if normal matched samples are not available. Meanwhile, some existing methods regard the whole RD profile as a Markov process and predict copy number states based on Markov models, such as m-HMM [17], PSE-HMM [34], and XHMM [35]. Considering the fact that CNV regions usually account for a small part of the whole genome, CNV_IFTV generates an anomaly score to evaluate RD values based on the isolation forest algorithm [30], i.e., it not only considers the variance of RD values across the genome to be analyzed but also the density of RD values. Apart from the global view on the RD profile, there exists one method developed by us previously that looks at the RD profile from a local perspective, CNV-LOF [31]. CNV-LOF regards CNVs as local outliers among all the RD values. Such an idea is helpful to detect less significant CNVs. All in all, the existing methods have their own characteristics and advantages under different applications. However, some realistic scenarios should be further addressed for the improvement of CNV detection. For instance, the observed RD profile is relatively crude due to the contamination of normal genomes in tumor genomes and bias in sequencing reads. Moreover, almost all of the existing methods deal with the RD profile in a line shape. This may lead to unfairness between the genome ends and the middle part of the genome.

With careful consideration of the issues above, in this paper, we developed an alternative approach, called CIRCNV, for the detection of CNVs from NGS data. The CIRCNV method is different from the existing methods. It transfers the read depth profile from a line shape to a circular shape via a polar coordinate transformation. Such nonlinear transformation can generate a meaningful two-dimensional dataset for CNV analysis and can promote fairness between the ends and middle part of the genome to be analyzed. For the evaluation of the transformed RD values, similar to the strategy of CNV-LOF, CIRCNV takes a local view on the RD profile and calculates a local outlier factor score for each genome region. To mitigate the influence of normal genome contamination, CIRCNV estimates tumor purity for each analyzed sample based on first-round detected deletions and then recovers the truth RD profile. With the truth RD profile, a second-round CNV declaration is carried out for the improvement in performance. To test CIRCNV, we generated a great number of simulated datasets based on real sequencing data for conducting experiments and performed comparisons with other peer methods in terms of sensitivity and precision. Furthermore, we applied the CIRCNV method to a set of real sequencing samples for the validation of the performance. The results indicate that CIRCNV is valuable and can find out biologically relevant events.

The remainder of this paper is organized as follows. Section 2 describes the flowchart of CIRCNV and its major principles. Section 3 presents the experimental results on simulation and real sequencing datasets and discusses the performance of CIRCNV in comparison with other methods. In Section 4, we conclude the proposed method and provide an outline of future work.

## 2. Materials and Methods

### 2.1. Overview of CIRCNV

**Figure 1:**
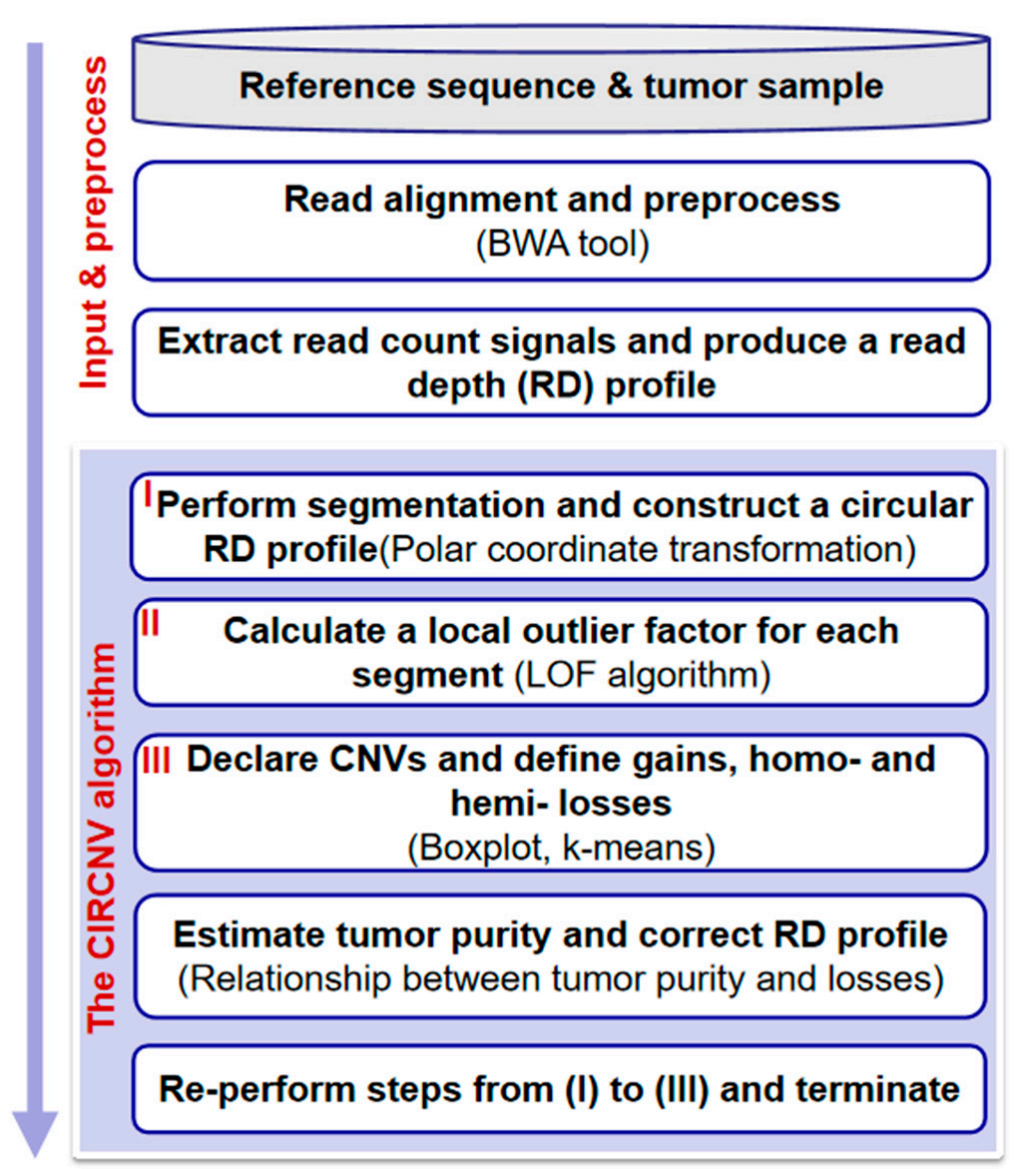
Flowchart of the CIRCNV method. It is composed of two parts: input and preprocessing, and the algorithm of CIRCNV.

The flowchart of the CIRCNV method is depicted in Figure 1. It starts with the input of an RD profile of one tumor sample and goes through three major steps to discover CNVs. These steps are re-performed to improve the accuracy of CNV detection by carrying out an estimation of tumor purity and a correction of the RD profile. These steps include: (1) performing a segmentation process on the observed RD profile and constructing a circular RD profile by using polar coordinate transformation; (2) calculating a local outlier factor for each segment; (3) declaring CNVs and defining gains and homogeneous (homo-) and heterogeneous (hemi-) losses. In the following text, we present a detailed description of the input RD profile, the aforementioned four steps, estimation of tumor purity, and correction of the RD profile.

### 2.2. Input and Preprocessing

The input files include a reference sequence and a tumor sample to be analyzed. The reference sequence could be selected from the commonly used version, human genome 19. The sequencing reads of the tumor sample are aligned to the reference genome by using one of the classic tools, BWA [36]. The resulting alignment file could be further handled by using SAMtools [37] to obtain a read count (RC) profile. Due to the existence of the symbol “N” in the reference genome, there will be no reads to be aligned on such positions. Zero RC values on such positions may mislead the analysis of the whole RC profile, since such values may be mistaken as deletion events from the perspective of fluctuation of RC values. Thus, we skip the “N” positions before starting the detection of CNVs from the RC profile.

Subsequently, we divide the RC profile into non-overlapping and continuous genome bins of the same size (e.g., 1000 base pairs) and then calculate a read depth (RD) value for each genome bin by averaging the RC values among the positions with the genome bin. Thus, an RD profile can be achieved. With this, we further carry out a GC content bias correction process to generate reasonable input data for the detection of CNVs. This process can be found in [24,38,39].

### 2.3. Performing Segmentation and Constructing a Circular RD Profile

With the preprocessed RD profile, we adopt the circular binary segmentation (CBS) algorithm [39] to perform a segmentation process. The purpose of such segmentation is to generate a segment-based unit (i.e., a genome region composed of a set of adjacent genome bins) for the subsequent analysis of CNVs. The segment-based unit is generally more reasonable than the genome bin-based unit since adjacent genome bins are usually and intrinsically correlated [31]. Thereby, a segment-based RD profile can be obtained, in which the RD value of each segment is the averaged RD value among the bins within the segment.

For convenience, we denote the segment-based RD profile as $S^{\prime }=\left\{s_{1}^{\prime },\;s_{2}^{\prime },\;\ldots ,s_{n}^{\prime }\right\}$, where n represents the total number of segments and $s_{i}^{\prime }$ represents the *i*-th segment. $s_{i}^{\prime }$ is composed of a two-tuple ($p_{i}^{\prime },r_{i}^{\prime }$), where $p_{i}^{\prime }$ represents the segment position (i.e., index) and $r_{i}^{\prime }$ represents the RD value of the *i*-th segment. We then adopt a polar coordinate transformation to transfer $S^{\prime }$ from a line shape to a circular-shaped RD profile, denoted as $S=\left\{s_{1},s_{2},\;\ldots ,s_{n}\right\}$. Similarly, $s_{i}$ is composed of a two-tuple ($p_{i},r_{i}$). The transformation formula is expressed as shown below.

(1) $$\{\begin{array}{c} p_{i}=r_{i}^{\prime }\times \cos\theta \\ r_{i}=r_{i}^{\prime }\times \sin\theta \end{array}$$

where θ is calculated for each segment by the following formula.

(2) $$\theta =2\pi \frac{p_{i}^{\prime }-\min\left\{p_{1}^{\prime },\;p_{2}^{\prime },\;\;\ldots ,p_{n}^{\prime }\right\}}{\max\left\{p_{1}^{\prime },\;p_{2}^{\prime },\;\;\ldots ,p_{n}^{\prime }\right\}-\min\left\{p_{1}^{\prime },\;p_{2}^{\prime },\;\;\ldots ,p_{n}^{\prime }\right\}}$$

where max{.} and min{.}  denote the maximum and minimum position indices, respectively.

**Figure 2:**
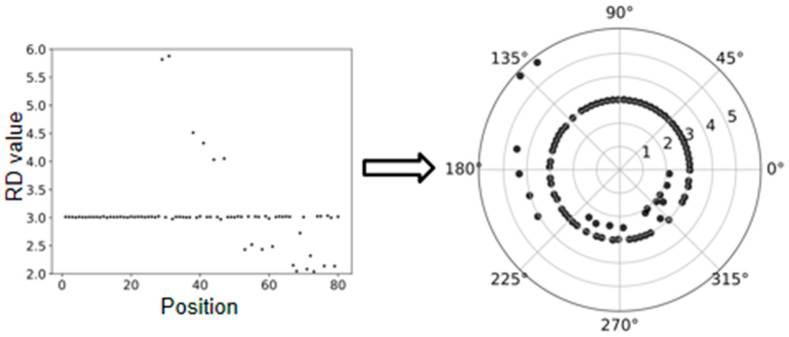
An example to show the polar coordinate transformation from a line-shaped RD profile to a circular-shaped RD profile.

For a clear understanding of the transformation, we provide an example in Figure 2, where the outlier elements above the line are mapped to the outside of the circle and the outlier elements under the line are mapped to the inside of the circle. This circular-shaped transformation could lead to two aspects of effects. First, a meaningful two-dimensional dataset can be obtained for the analysis of CNVs, since CNVs can be regarded as outliers from the cluster of normal genome segments and can be reasonably observed on a two-dimensional distributed space. Second, such transformation can promote fairness between the ends and middle part of the genome, since the number of segments around the elements towards the ends is less than that around the elements towards the middle part of the genome in the line-shaped RD profile, while the circular-shaped RD profile can avoid this issue.

### 2.4. Calculating Local Outlier Factors for Each Segment

For segment $s_{i}$ denoted by ($p_{i},r_{i}$), we calculate a local outlier factor LOF($s_{i}$) as the anomaly score. This calculation involves a distance parameter *k*, which is explained as the *k*-distance neighborhood of segment $s_{i}$ to segment *o*. In other words, segment *o* represents the *k*-th nearest segment to $s_{i}$ among all the segments in S, except $s_{i}$ itself [40]. The value of this parameter can explain how isolated $s_{i}$ is from its surrounding neighborhoods and thus can be used to find local CNVs [31]. Local CNVs are also called focal CNVs and are very common in the human tumor genome. Accurate identification of such CNVs is a crucial step in the analysis of tumor mechanisms and finding target cancer drugs. Thus, using the anomaly score LOF($s_{i}$) with a suitable parameter value of *k* can facilitate the identification of local CNVs. The formulas related to the calculation of LOF($s_{i}$) can be found in [31] and are not re-listed here. In our experiments, the parameter value of *k* was empirically set to 10.

### 2.5. Declaring CNVs and Defining Gains and Homo- and Hemi-Losses

With the anomaly score LOF($s_{i}$) for segment $s_{i}$, how to determine whether $s_{i}$ is a CNV or not is a crucial step. Generally, the larger the value of LOF($s_{i}$), the more likely segment $s_{i}$ is a CNV. However, setting a cutoff threshold for the LOF($s_{i}$) is not an easy task since different samples may produce different distributions of LOF values and there is no prior knowledge to supervise us to choose a suitable value for the cutoff threshold. Based on the viewpoint that most segments in the tumor genome are normal, i.e., most of the LOF values are at a limited range, it is meaningful and feasible to find out the abnormal LOF values via analyzing the distribution of LOF values. Here, we choose the boxplot procedure for this analysis [31], since it can produce a reasonable representation of the LOF value distribution and can use a general cutoff threshold for the extraction of abnormal LOF values. Then, the segments with the abnormal LOF values are regarded as CNVs. For related details, readers are suggested to refer to [31].

Subsequently, we classify the CNVs into gains and losses by comparing their RD values to the mode of them. Specifically, if $s_{i}$ has been declared as a CNV and its RD value $r_{i}$ is larger than the mode, then it is defined as a gain; otherwise, it is a loss. For the losses, we further perform a classification into hemi-losses and homo-losses. Hemi-loss means that one of the diploids (two copies) is deleted, and homo-loss means that both copies are deleted. Here, it is not easy to define a threshold for this classification, since many artifacts such as noises and mapping errors can pose a significant influence. Instead, we adopt the k-means algorithm for this classification. For convenience, the classified losses are denoted by $X=\{x_{1},x_{2},\ldots ,x_{k_{1}}\}$ for hemi-losses and $Y=\{y_{1},y_{2},\ldots ,y_{k_{2}}\}$ for homo-losses, where $k_{1}$ is the number of hemi-losses and $k_{2}$ is the number of homo-losses.

### 2.6. Estimating Tumor Purity and Correcting the RD Profile

Since the tumor tissues to be sequenced usually contain a fraction of normal cells and such contamination can pose a great influence on the detection of variations [41], making a reasonable estimation of tumor purity can help to correct the observed signals from tumor samples to be analyzed. Therefore, in this section, we use the observed RD profile and losses from the tumor sample to estimate tumor purity and then perform a correction to the RD profile. The purpose of this step is to provide a relative truth RD profile for CNV detection. Specifically, we can use the two types of losses to establish equations between tumor purity (α), observed RD values, and absolute RD values. The equations are expressed as below.

(3) $$\{\begin{array}{c} r\left(x_{i}\right)=\frac{\bar{r}}{2}\alpha +\bar{r}\left(1-\alpha \right)\\ r\left(y_{i}\right)=\bar{r}\left(1-\alpha \right) \end{array}$$

where $r\left(x_{i}\right)$ and $r\left(y_{i}\right)$ represent the observed RD values of the hemi-loss segment $x_{i}$ and homo-loss segment $y_{i}$, respectively, $\bar{r}$ represents the RD value corresponding to a normal segment, and $\frac{\bar{r}}{2}$ can be explained as the absolute RD value of hemi-loss.

With the above equations, the tumor purity (α) can be calculated given $r\left(x_{i}\right)$, $r\left(y_{i}\right)$, and $\bar{r}$. Then, each hemi-loss or homo-loss can produce a value for α, and a total of ($k_{1}$+$k_{2}$) values can be derived. Considering that the observed RD values can be affected by sequencing and mapping uncertainties, there exist some differences between the derived values for α. To obtain a relatively reasonable estimation value, we average all the ($k_{1}$+$k_{2}$) derived values and determine a final value of α.

With the estimated tumor purity α above, we perform a correction to the observed RD profile $S^{\prime }=\left\{s_{1}^{\prime },\;s_{2}^{\prime },\;\ldots ,s_{n}^{\prime }\right\}$ in order to obtain a less biased RD profile for the tumor samples to be analyzed. The correction formula is expressed as below.

(4) $$r_{i}^{\prime }=r_{i}^{\prime \prime }\alpha +\bar{r}\left(1-\alpha \right)$$

where $r_{i}^{\prime \prime }$ denotes the absolute RD value for the i-th segment $s_{i}^{\prime }$. Then, the value of $r_{i}^{\prime \prime }$ can be derived by changing the formula above. Thus, an updated RD profile can be achieved by using $r_{i}^{\prime \prime }$ to replace $r_{i}^{\prime }$. With this updated RD profile, a second-round CNV detection process will be carried out.

### 2.7. Core Algorithm of CIRCNV

For a clear understanding of the principle and procedure of the CIRCNV method, we demonstrate its core algorithm using a set of steps in Algorithm 1.
**Algorithm 1** The core algorithm of CIRCNV(1) Input data: an observed RD profile; (2) Performing segmentation on the input RD profile and obtaining a segment-based RD profile $S^{\prime }$; (3) Performing the polar coordinate transformation and obtaining a circular-shaped RD profile S; (4) Calculating a LOF value for each segment $s_{i}$, $s_{i}\in S$; (5) Declaring CNVs via boxplot procedure and defining gains, hemi-losses X, and homo-losses Y; (6) Estimating tumor purity α by using X and Y, and updating $S^{\prime }$; (7) Re-performing steps (3) to (5); (8) Outputting the final results (gains and losses).

## 3. Results and Discussion

With the principle of CIRCNV described above, we used the Python language to implement it and develop the corresponding software package. The software package is freely available at (https://github.com/BDanalysis/CIRCNV, accessed on 10 June 2021) and can be implemented easily by referring to its manual. To test the performance of the CIRCNV method, we first carried out a large number of experiments via simulation studies, since simulation studies can provide the absolute ground truth for the quantification of performance [41,42], and then we applied the proposed method to analyze several real tumor samples for showing its usefulness. In both simulation and real sequencing sample experiments, we made comparisons between CIRCNV and several peer methods in terms of sensitivity, precision, F1-score, or overlapping density score. For a fair comparison, we used a constant parameter value in the CIRCNV algorithm and used the default parameter values in the peer methods during the experiments of their algorithms and the reliability of the proposed method. Aiming at this point, simulation and real experiments were conducted. A simulation experiment is an effective and objective evaluation strategy, which can provide a comparison criterion to quantify the performance of the proposed method. In the simulation experiment, three popular published algorithms (BIC-seq2, SeqCNV, and CNVkit) that can be used to effectively detect matched case–control samples were selected for comparison with CBCNV. The performances of these methods are evaluated from three perspectives. First, the sensitivity and false discovery rate (FDR) of the four methods are evaluated at six CNV size levels. Then, the sensitivity and FDR of each method in the CNV gain and loss regions are analyzed and discussed. Finally, three indicators (recall, precision, and F1-score) are used to comprehensively evaluate the performance of each method. In real data applications, the proposed algorithm was used to detect two pairs of matched breast cancer WGS samples. As the ground truths of the real datasets are unknown, the number of overlapping CNV events and the number of predicted CNV events were adopted to evaluate the performance of each method. To further verify the performance of the proposed method, we used the overlapping density score method to quantify the performance of each method. The experimental results demonstrate that CBCNV is a powerful CNV detection tool.

### 3.1. Simulation Studies

The first step of carrying out simulation studies is to produce simulation data. Currently, there are a number of simulation tools that can be used to generate NGS data and simulate genomic variations. Here, we adopted our previously developed simulation algorithm IntSIM [41] to imitate CNVs and generate sequencing reads for tumor samples. The tumor purity was set to range from 0.2 to 0.3 in our experiments since such small tumor purity values are a frequent phenomenon in the real world and provide a challenge for CNV detection methods. Thus, testing and comparing our method to peer methods on the sequencing data with such small tumor purity are meaningful. For the sequencing coverage depth, we set it to a moderate value of 6x. In each simulation configuration, we generated fifty replications for testing the methods. A detailed description of the simulation process can be found in [31,41].

**Figure 3:**
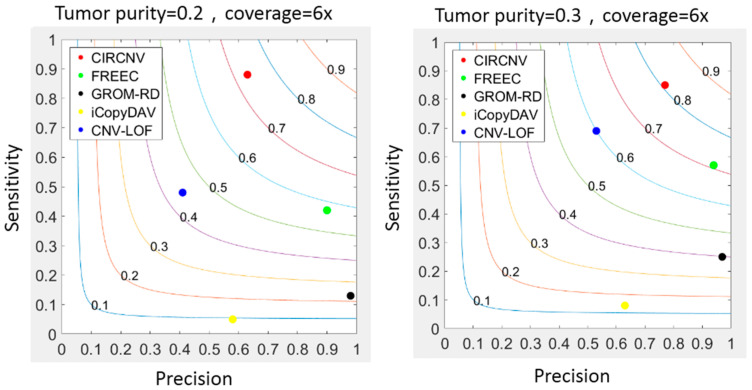
Comparisons between the CIRCNV method and four peer methods with respect to sensitivity, precision, and F1-score on simulation data. The color curves ranging from 0.1 to 0.9 denote the F1-scores.

With the simulation datasets above, we implemented the CIRCNV method and four peer methods including FREEC [25,43], GROM-RD [33], iCopyDAV [29], and CNV-LOF [31]. The comparisons between these five methods were made with respect to sensitivity, precision, and F1-score. The F1-score is the harmonic mean of sensitivity and precision and can be explained as the tradeoff between them. The sensitivity is calculated as the number of correctly declared CNVs divided by the total number of ground truth CNVs, and the precision is calculated as the number of correctly declared CNVs divided by the total number of declarations. Here, one correctly declared CNV is termed when it becomes overlapped with markers from the region of one ground truth CNV. The performance comparison results are shown in Figure 3, where the sensitivity and precision values are the average values of the fifty simulation replications. From the comparative results, we can notice that CIRCNV obtains the largest sensitivity value, followed by CNV-LOF, FREEC, GROM-RD, and then iCopyDAV. As for the precision, GROM-RD ranks first, followed by FREEC, CIRCNV, iCopyDAV, and then CNV-LOF. Generally, the values of sensitivity and precision influence each other under a constant experimental situation. Thus, the tradeoff between sensitivity and precision can account for the performance reasonability. In terms of the F1-score, CIRCNV performs superiorly, and FREEC ranks second, followed by CNV-LOF, GROM-RD, and then iCopyDAV. Therefore, we may conclude that the CIRCNV method displays the best performance among the five methods in these simulation experiments.

Furthermore, we may note that the sensitivity, precision, and F1-score of almost all five methods are improved when the tumor purity level in the simulation data is increased. For example, the F1-score of CIRCNV is around 0.7 in the simulation configuration of tumor purity of 0.2, and it reaches over 0.8 under tumor purity of 0.3. This is because a large tumor purity value can increase the signal ratio of the tumor genome to the normal genome and thus facilitate the detection of CNVs. More analysis results about the tumor purity influence on CNV detection can be found in [41].

The comparison of memory and running time of some of the simulation data and the precision and sensitivity of the CIRCNV and CNV-LOF (1x simulation datasets) are presented in the Supplementary Materials.

**Figure 4:**
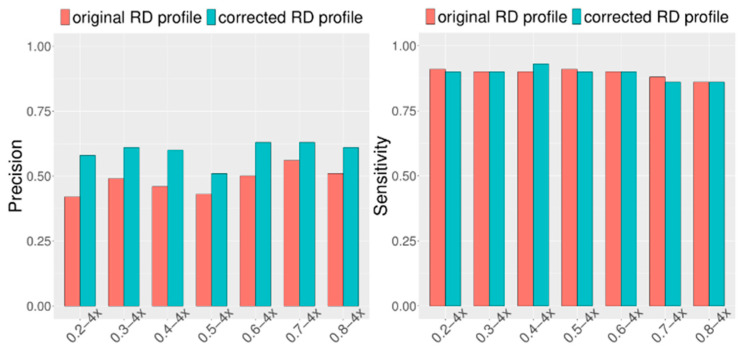
Performance comparisons between the first-round (using original RD profile) and the second-round (using corrected RD profile) CNV detected by the CIRCNV method with respect to sensitivity and precision. The simulation tumor purity ranges from 0.2 to 0.8, and the sequencing coverage depth is 4×.

**Figure 5:**
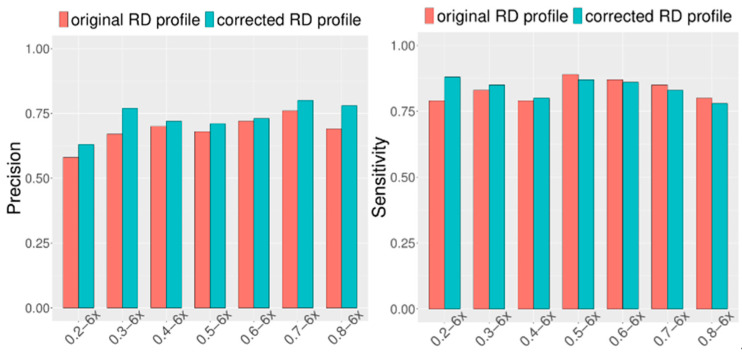
Performance comparisons between the first-round (using original RD profile) and the second-round (using corrected RD profile) CNV detected by the CIRCNV method with respect to sensitivity and precision. The simulation tumor purity ranges from 0.2 to 0.8, and the sequencing coverage depth is 6×.

Considering that CIRCNV carried out two rounds of CNV detection based on the original RD profile and corrected RD profile, it is necessary and meaningful to observe how much improvement can be achieved in the second round compared to the first round. For this purpose, we extended the simulation datasets by setting the configuration as follows: tumor purity range from 0.2 to 0.8 and sequencing coverage depth range from 4x to 6x. At the same time, in each of the configurations, fifty replications were produced for demonstrating the stability of the performance. We ran the CIRCNV algorithm on these datasets and made a comparison between the first round by using the original RD profile and the second round by using the corrected RD profile in terms of sensitivity and precision. The comparative results are shown in Figure 4 and Figure 5. From the figures, we can observe that the precision is undoubtedly improved in the second CNV detection round by using the corrected RD profile relative to the first CNV detection round by using the original RD profile, while the sensitivity is not changed obviously. This could be explained as follows. The correction of the RD profile can enhance the difference between CNV regions and normal copy number regions. Such differences can help in the discrimination of CNVs from normal regions. From both Figure 4 and Figure 5, we can note that the precision is obviously improved when the sequencing coverage depth increases from 4× to 6×. Nevertheless, the sensitivity is slightly decreased from 4× to 6×. Such phenomena might be explained as follows. The larger coverage depth may bring about more data noise, while our proposed method tends to regard the noise and the true CNVs with a similar RD to the noise from normal events. Thus, CIRCNV can obtain a high precision value at the cost of sensitivity.

### 3.2. Detection of Copy Number Variants from Breast Cancer Sample

To further validate the performance of the CIRCNV method, we applied it to analyze three real sequencing tumor samples on a whole genome. These samples were downloaded from the European Genome-phenome Archive (EGA) data project at (https://ega-archive.org/, accessed on 9 June 2021). These samples were sequenced from ovarian cancer patients numbered with EGAR00001004802_2053_1 and EGAR00001004836_2561_1, and one lung cancer patient numbered with EGAD00001000144_LC.

**Figure 6:**
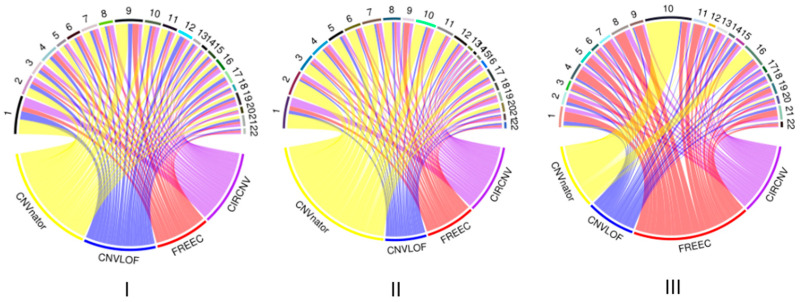
Result comparison of the four methods on the whole-genome sequencing data from samples of EGAR00001004802_2053_1 (**I**), EGAR00001004802_2053_1 (**II**), and EGAD00001000144_LC (**III**). The distributions of the numbers of CNVs detected by the four methods and the numbers of detected CNVs in each autosome chromosome.

**Figure 7:**
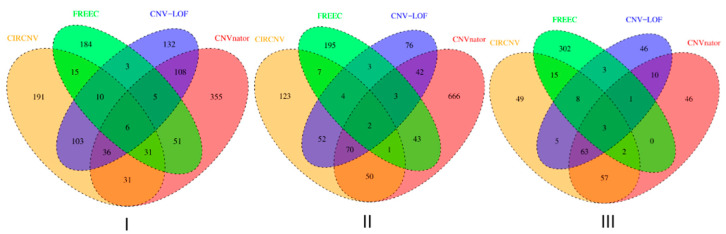
Venn diagrams to show the overlapping and non-overlapping CNVs between each pair of methods for each of the three tumor samples, EGAR00001004802_2053_1 (**I**), EGAR00001004836_2561_1 (**II**), and EGAD00001000144_LC (**III**).

We performed the CIRCNV method on the whole genome including twenty-two autosome chromosomes and compared it to three peer methods, FREEC, CNVnator, and CNV-LOF. The comparative results are shown in Figure 6 and Figure 7 for the three samples. These figures show the number of declared CNVs in each of the autosome chromosomes. We can notice that CNVnator obtains the largest number of CNVs, followed by CIRCNV, CNV-LOF, and FREEC. However, the number of declarations cannot account for the merits of the methods. Due to the lack of answers in real tumor samples, it is not easy to estimate the sensitivities and precisions for the methods. Instead, we use Venn diagrams to describe the overlapping among different methods in Figure 6 and Figure 7 for the two samples. From these figures, we may observe that CIRCNV obtains the largest number of CNVs overlapped with other methods, although it does not obtain the largest number of declarations. This means that CIRCNV might be more powerful than other methods since such overlapped CNVs are generally more likely to be the actual CNVs than the non-overlapping CNVs.

To quantitatively evaluate the overlapped number of CNVs among different methods, we adopted our previously proposed measurement overlapping density score (ODS) [6] for this evaluation. The formula of calculating the ODS is described in detail in [6] and is not listed here for simplicity. The larger the ODS value to be achieved by one method, the better the performance of that method. For the three tumor samples of EGAR00001004802_2053_1 (4802_2053_1), EGAR00001004836_2561_1 (4836_2561_1), and EGAD00001000144_LC (0144_LC), the ODS values for the four methods are listed in Table 1, where we can see that CIRCNV and CNV-LOF obtained the largest ODS values. Here, it should be noticed that the ODS value takes into account both the number of overlapped CNVs and the number of called CNVs and is dependent on the samples to be analyzed. For one sample, the larger the ODS value, the better the method. For different samples, the ODS values obtained by one method are not comparable.

For the CNVs detected by the CIRCNV method, we further find that these CNVs contain many biological genes associated with ovarian cancer. For instance, the CNVs detected in sample EGAR00001004802_2053_1 contain cancer-associated genes such as ELOVL7 [10] located at 5q12.1, IFNB1 [44] located at 9p21.3, BAG1 [45] located at 9p21.1, RECK [46] located at 9p13.3, DAPK1 [47], CTSL [48], and CCRK [49] located at 9q21.33, SYK [50] located at 9q22.2, and NR4A3 [51] and TMEFF1 [52] located at 9q22.33. Similarly, the CNVs detected in sample EGAR00001004836_2561_1 also contain many cancer-associated genes, such as CD80 [53], GSK3B [54], FSTL1 [55], CASR [56], PARP9 [57], and PARP15 [58] located at 3q13.33. Additionally, the CNVs detected in sample EGAD00001000144_LC(0144_LC) contain a great number of cancer genes, including B3GALT6 [59] located at 1p36.33, DVL1 [60] located at 1p36.33, and NOTCH2NL [61] located at 1q21.1. Thus, we can conclude that CIRCNV is practical in the application to real sequencing samples for CNV detection.

## 4. Conclusions

In this paper, we proposed an alternative method, called CIRCNV, for the detection of CNVs in sequencing data. This method is an extended version of our previously developed method CNV-LOF. Both CIRCNV and CNV-LOF use the local outlier factor as the measurement for the prediction of CNVs. The difference of CIRCNV from CNV-LOF lies in its two new features: (1) it transfers the read depth profile from a line shape to a circular shape via a polar coordinate transformation, in order to improve the efficiency of the read depth (RD) profile; and (2) it performs two rounds of CNV declaration via estimating tumor purity and recovering the truth circular RD profile.

We tested and evaluated the performance of CIRCNV via conducting simulation studies and real sequencing tumor sample applications. The results from simulation studies demonstrate that CIRCNV outperforms peer methods with respect to sensitivity, precision, and the F1-score. We can also observe that the second round of CNV detection in the CIRCNV algorithm is meaningful due to the obvious improvement in precision. The results from real sample applications illustrate that CIRCNV obtains the largest number of consistent CNVs with peer methods and can detect biologically meaningful CNVs. Therefore, CIRCNV can be expected to be a reliable tool in the field of analyzing CNVs in tumor genomes.

In future work, we intend to extend the current version of the CIRCNV method from the two following perspectives. First, we will integrate the detection of somatic nucleotide variations (SNV) [62] into the CNV detection process, since both of these types of genomic mutations frequently and concurrently appear in the human genome. The integrated detection of multiple types of genomic mutations will be more efficient than a single type of genomic mutation analysis. Second, we plan to combine the information of split reads into the detection of CNV contents, in order to improve the detection of boundaries of CNVs.

## Figures and Tables

**Table 1:** Comparison of ODS values between the four methods on the two tumor samples.

	CIRCNV	CNVnator	FREEC	CNV-LOF
4802_2053_1	27.07	15.83	11.03	21.00
4836_2561_1	25.16	10.55	1.73	27.55
0144_LC	29.6	16.6	0.73	23.3

## Data Availability

Publicly available datasets were analyzed in this study: ovarian cancer patients numbered with EGAR00001004802_2053_1 and EGAR00001004836_2561_1, and one lung cancer patient numbered with EGAD00001000144_LC. and they were downloaded from the European Genome-phenome Archive (EGA) data project at (https://ega-archive.org/, accessed on 9 June 2021).

## Supplementary Materials

The following are available online at https://www.mdpi.com/article/10.3390/biology10070584/s1, Table S1: The comparison of running time and memory usage between CIRCNV and CNV-LOF under the simulation configuration of tumor purity of 0.2 and coverage depth of 4×. Table S2: The comparison of running time and memory usage between CIRCNV and CNV-LOF under the simulation configuration of tumor purity of 0.2 and coverage depth of 6×. Table S3: The comparison of running time and memory usage between CIRCNV and CNV-LOF under the simulation configuration of tumor purity of 0.3 and coverage depth of 4×. Table S4: The comparison of running time and memory usage between CIRCNV and CNV-LOF under the simulation configuration of tumor purity of 0.3 and coverage depth of 6×. Table S5: The comparison of precision and sensitivity between CIRCNV and CNV-LOF when running on sequencing data with tumor purity of 0.8 and coverage depth of 1×.
