# Supplementary material for: CIRCNV: Detection of CNVs Based on a Circular Profile of Read Depth from Sequencing Data
Source: Biology (Basel). 2021 Jun 25;10(7):584. doi: 10.3390/biology10070584 (PMC8301091; doi:10.3390/biology10070584)
Supplement: Supplementary file 1 [file biology-10-00584-s001.zip › biology-1216408-supplementary.pdf]

1. The comparison of running time and memory between CIRCNV and CNV-LOF.

We implement the proposed method and the CNV-LOF method on simulation data with various configurations, and calculate the corresponding running times and memory usage. The comparative results are shown in Tables s1 to s4.

Table S1 The comparison of running time and memory usage between CIRCNV and CNV-LOF under the simulation configuration of tumor purity of 0.2 and coverage depth of 4x.

	Max_time(s)	Min_time(s)	Mean_time(s)	Max_mem(Mb)	Min_mem(Mb)	Mean_mem(Mb)
CIRCNV	21	19	20	738.8	729.43	732.04
CNV-LOF	20	19	19.76	738.52	729.45	733.00

Table S2 The comparison of running time and memory usage between CIRCNV and CNV-LOF under the simulation configuration of tumor purity of 0.2 and coverage depth of 6x.

	Max_time(s)	Min_time(s)	Mean_time(s)	Max_mem(Mb)	Min_mem(Mb)	Mean_mem(Mb)
CIRCNV	24	23	23.7	738.88	728.82	732.85
CNV-LOF	24	22	23.2	737.74	729.35	733.19

Table S3 The comparison of running time and memory usage between CIRCNV and CNV-LOF under the simulation configuration of tumor purity of 0.3 and coverage depth of 4x.

	Max_time(s)	Min_time(s)	Mean_time(s)	Max_mem(Mb)	Min_mem(Mb)	Mean_mem(Mb)
CIRCNV	22	19	19.2	738.66	728.59	730.38
CNV-LOF	21	19	20	738.5	729.39	730.07

Table S4 The comparison of running time and memory usage between CIRCNV and CNV-LOF under the simulation configuration of tumor purity of 0.3 and coverage depth of 6x.

	Max_time(s)	Min_time(s)	Mean_time(s)	Max_mem(Mb)	Min_mem(Mb)	Mean_mem(Mb)
CIRCNV	24	23	23.5	737.88	729.49	731.36
CNV-LOF	24	23	23.15	737.55	729.36	732.34

Where Max_time denotes the maximum running time among the fifty simulation replications in each simulation configuration, Min_time denotes the minimum running time among the fifty simulation replications in each simulation configuration, Mean_time denotes the average running time among the fifty simulation replications in each simulation configuration. Similarly, Max_mem, Min_mem, Mean_mem denote the corresponding memory required in running the software.

2. Experiment on sequencing data with low pass coverage depth

In order to test the performance of our proposed method in running on sequencing data with low pass coverage depth, we simulate a set of datasets with tumor purity of 0.8 and coverage depth of 1x, and carry out the CIRCNV and CNV-LOF methods on these datasets.

The comparative result is shown in Table s5.

Table S5 The comparison of precision and sensitivity between CIRCNV and CNV-LOF when running on sequencing data with tumor purity of 0.8 and coverage depth of 1x

	precision	sensitivity
CIRCNV	0.53098	0.367171
CNV-LOF	0.3279	0.371
